# Pilomatrixoma Presenting as a Rapidly Expanding Mass of the Infant Nasion

**Published:** 2015-12-19

**Authors:** Lauren C. Nigro, Christine E. Fuller, Jennifer L. Rhodes

**Affiliations:** Virginia Commonwealth University, West Hospital, Richmond, Virginia

**Keywords:** pilomatrixoma, hair diseases, skin neoplasms, infant, midline mass

## Abstract

**Objective:** Pilomatrixomas are benign neoplasms originating from the cells of hair follicles. They typically present as a slowly enlarging, solitary mass on hair-bearing areas of the head and neck. While a common childhood lesion, pilomatrixomas are unusual in infancy. Our objective is to present an atypical pilomatrixoma located on the midline nasion of an 11-month-old as such a lesion and its management has not been previously described. **Methods:** Despite preoperative diagnostic imaging, including computed tomography and magnetic resonance imaging, the diagnosis was not made until examination by pathology after complete surgical excision. We also completed a thorough review of the literature pertaining to pilomatrixomas, which is presented in a concise fashion. **Results:** Our patient's clinical presentation did not correlate with traditional descriptions in the literature, skewing preoperative diagnosis. However, surgical management was ultimately appropriate and effective. To date, the patient has not demonstrated evidence of recurrence. **Conclusion:** We believe that this is the first such reported presentation of a pilomatrixoma. Given its incidence, we encourage readers to consider this diagnosis when evaluating similar pediatric skin lesions of the head and neck. Complete surgical excision is the definitive treatment.

Pilomatrixomas are benign tumors arising from the uncontrolled proliferation of hair matrix cells. They typically present as a slowly enlarging, solitary mass, most commonly on the hair-bearing areas of the head and neck.^1^^-^3 While common as a childhood lesion, pilomatrixomas are unusual in infancy.^2^^,^^4^^-^6 Rapid enlargement is rarely reported. We present a unique case of a rapidly growing mass of the midline nasion on an 11-month-old that was identified as a pilomatrixoma after excision.

The differential diagnosis for an expanding, midline mass of the infant nasion includes dermoid or epidermoid cyst, glioma, encephalocele, or vascular anomaly.^5^^,^^7^ Rapid enlargement of a soft tissue in a child also raises concern for malignancies, such as sarcoma or lymphoma. We believe that this is the first reported such presentation of a pilomatrixoma.

## CASE PRESENTATION

**Figure 1:**
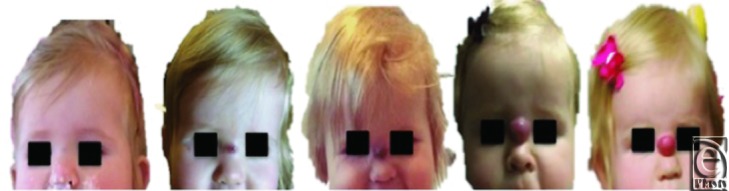
Preoperative lesion progression: progression of midline nasion lesion over 3 months as captured by the patient's mother/guardian. She was 11 months of age at the time of presentation when the last image in the sequence was taken.

A previously healthy, 11-month-old child developed a solid mass of the nasion. It began as a subtle swelling and rapidly expanded within 3 months, causing significant deformation of the child's face (Fig 1). The child's mother denied trauma to the area.

On examination, the mass was located directly between the orbits and measured approximately 17 mm in diameter. It appeared pink in color and did not transilluminate. It was nontender to palpation and had a rubbery texture. The remainder of the examination was unremarkable and the patient demonstrated no neurologic deficits.

The patient underwent surgical removal of the mass through a transverse, elliptical incision. The patient is doing well after 6 months of follow-up.

### Diagnostic Imaging

**Figure 2:**
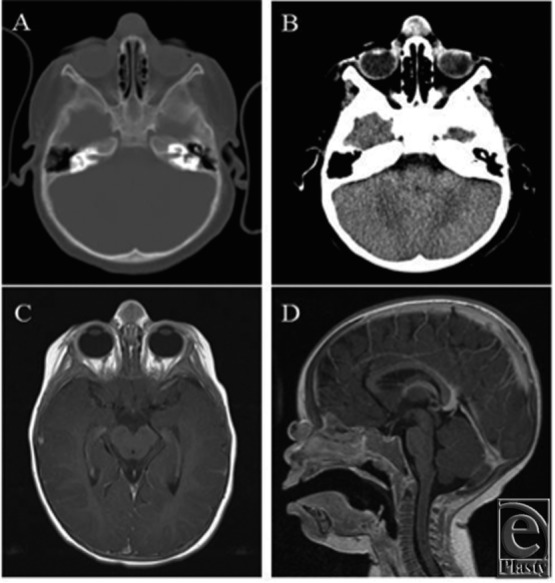
Diagnostic imaging: computed tomography of the head with contrast in the axial plane with arrows depicting the lesion in the (A) bone window and (B) brain window. Similarly, postcontrast magnetic resonance imaging of the head in the (C) axial plane, T1 and (D) sagittal plane, magnetization-prepared rapid gradient-echo (MP-RAGE).

Preoperative workup included computed tomography (CT) and magnetic resonance imaging (MRI) of the head. The CT scan demonstrated a soft tissue mass of mixed attenuation ventral to the nasal bone. Small portions of the mass demonstrated increased attenuation, consistent with calcification, surrounding a central portion with low attenuation. The mass did not appear to arise from the bone and did not demonstrate extension into the calvarial vault (Figs 2A and 2B). Magnetic resonance imaging demonstrated a solid mass overlying the nasion with small cystic components. The solid component demonstrated T1 isointense and T2 hypointense signals. There was rim enhancement as well as faint enhancement of the matrix. Notably, the midline structures were intact without intracranial or intranasal extension or involvement of underlying bone or adjacent tissues (Figs 2C and 2D).

### Pathology

**Figure 3:**
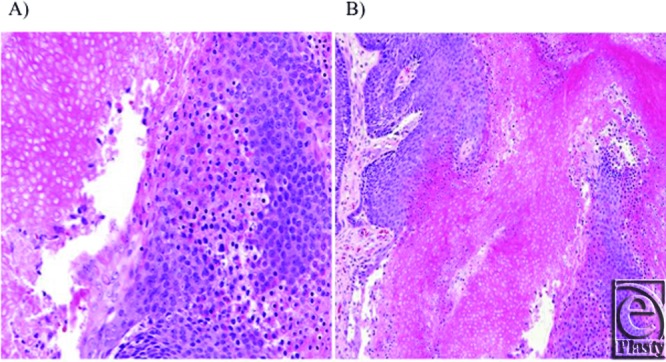
Pathology: Low- (A) and high-power (B) photomicrographs of pilomatrixoma showing solid sheets of basaloid cells with bland, round nuclei encircling islands of “ghost cells” with abundant eosinophilic cytoplasm lacking nuclei (hematoxylin and eosin stain, ×100 and ×200, respectively).

Surgical pathology revealed a pilomatrixoma. The lesion was well-demarcated, composed of solid sheets of basaloid cells with bland, round nuclei. Islands of “ghost cells” with abundant eosinophilic cytoplasm devoid of nuclei were present centrally, arising as abrupt keratinization from the surrounding basaloid cells. There was no evidence of significant nuclear atypia or mitotic activity, and calcification was not present (Fig 3).

## DISCUSSION

Pilomatrixoma, formerly referred to by the eponym calcifying epitheliomas of Malherbe, were first described in 1880 by Malherbe and Chenantais.^8^ This tumor represents 0.001% to 0.003% of all dermatopathologic specimens.^9^ While up to 70% arise in the head and neck region, pilomatrixomas over the nose are rare.^2^^,^^4^^-^6^,^^10^

These tumors typically present as asymptomatic, slow-growing, blue-colored, subcutaneous or intradermal, firm nodules.^6^ When the skin overlying the tumors is stretched or placed under perpendicular tension, it can angulate or fold in a specific manner that has been referred to as the “Tent Sign” or “Skin Crease Sign.”^11^

Given their cells of origin, pilomatrixoma have a predisposition toward hear-bearing areas, particularly of the head and neck region. Age at presentation typically displays a bimodal pattern with peaks seen in the second and sixth decades of life.^12^ Presentation in infancy is uncommon.^6^

The etiopathogenesis of pilomatrixomas remains unknown, although investigations have demonstrated an association with genetic mutations in β-catenin and similar tumors have been induced by the polyomavirus.^13^^-^15 There is also an association of pilomatrixoma with certain genetic disorders, such as Rubinstein-Taybi syndrome, myotonic dystrophy, Turner syndrome, Gardner syndrome, Churg-Strauss syndrome, xeroderma pigmentosum, sarcoidosis, and basal cell nevus syndrome.^16^^-^24

In our patient, the pilomatrixoma was rapidly growing, pink-colored, and located along the midline of her nasion. This unique presentation obscured the diagnosis, which is not uncommon for pilomatrixoma.^5^ Previous studies have noted a correct diagnosis in only 12.5% to 55% of cases.^4^^,^^10^^,^^25^^,^^26^ This low diagnostic accuracy may be attributed to a variety of factors such as lack of awareness of the tumor or unusual presentation (as in the case of our patient) mimicking that of another lesion. Therefore, the diagnosis of pilomatrixoma based solely on patient history and examination is difficult as these lesions are often confused with dermoid or epidermal cysts, brachial cleft remnants, vascular lesions, or malignant tumors.^27^

A rapidly expanding mass over the midline of the nasion in an infant typically suggests a differential diagnosis of dermoid or epidermoid cyst, nasal encephalocele or glioma, or vascular tumor or malformation.

Dermoid cysts are the most common congenital midline mass, resulting from failure of neuroectodermal structures to regress during embryologic development. They are lined with keratinized squamous epithelium and contain mesodermal adnexal structures. They can present as a pit, fistula, or noncompressible mass, commonly along the midline, anywhere from the glabella to the columella, by 2 to 3 years of age.^28^ Features that distinguish nasal dermoid cysts from pilomatrixoma include sebaceous discharge or protrusion of hair from a sinus or punctum and intracranial extension in up to 45% of cases. 29^-^32 The entire cyst and sinus tract, if present, must be excised to prevent recurrence.

Encephaloceles, meningoceles, and meningoencephaloceles are rare, extracranial herniations of brain, meninges, or both caused by a failure of the surface ectoderm to separate from the neuroectoderm during development. Nasal encephaloceles, which include frontoethmoidal and basal encephaloceles, represent approximately 15% of encephaloceles. While they may be due to structural weakness or arrested growth of the frontal and ethmoidal bones, hyperthermia, viral infections, exposure to teratogens, or in utero folic acid deficiency, the exact etiology remains unclear.^33^^-^35 They typically present at birth as compressible, soft, blue- or skin-colored masses or protrusions overlying the nose, glabella, or forehead that leak cerebrospinal fluid and enlarge when the child cries or with compression of the internal jugular veins (Furstenberg test).^34^^,^^36^ Unlike pilomatrixoma, nasal encephaloceles often appear intranasally or within the nasal cavity, causing upper airway obstruction. Children of Southeast Asian origin are the most frequently affected (1 in 5000-6000).^34^^,^^37^ Nasal encephaloceles are commonly associated with hydrocephalus, corpus callosum agenesis, hypertelorism, cleft palate, and other midline defects.^34^ As with pilomatrixoma, surgical resection is the only effective treatment and, given the connection with the central nervous system, requires a multidisciplinary team.

Imaging modalities such as ultrasonography, CT, or MRI can aid in the diagnosis of pilomatrixomas. In one retrospective review comparing pilomatrixomas with other subcutaneous growths, Choo et al^38^ found the following sonographic features to be useful in distinguishing pilomatrixomas from nonpilomatrixoma: heterogeneous echotexture, scattered-dot patterns of internal echogenic foci (consistent with internal calcification), hypoechoic rim, and posterior shadowing. These findings have been described by others as well.^39^^-^41 In another retrospective review, Hwang et al^40^ found that when ultrasonography was used, a correct preoperative diagnosis was made in 76% of cases versus 33% using clinical findings only. Similarly, Lim et al,^41^ in a review of the radiologic characteristics of pilomatrixomas, found that the correct preoperative diagnosis was made in 82%, 87%, and 60% of cases evaluated by ultrasonography, CT, and MRI, respectively.

On CT scan, pilomatrixomas typically appear as well-defined, heterogeneous, subcutaneous masses with calcific foci, microcalcifications, or complete calcification. They are isodense with surrounding tissue prior to contrast and demonstrate mild to moderate enhancement with contrast. On magnetic resonance image, pilomatrixoma demonstrates internal reticulations, patchy areas of increased signal intensity, and rim enhancement with gadolinium-enhanced T1-weighted and T2-weighted imaging. They are isodense with surrounding tissue on T1-weighted imaging alone.^41^ Our patient's pilomatrixoma was similarly well-defined and confined to the superficial soft tissues with portions of mixed attenuation, small calcifications, and matrix and rim enhancement, consistent with the findings described by Lim et al^41^ in 2007.

Pilomatrixomas are composed of a thin layer of peripherally located basaloid cells and centrally located, clusters of anucleated cells with eosinophilic cytoplasm, also known as ghost or shadow cells, initially described in 1944 by Highman and Ogden.^42^^,^^43^ In addition, foreign body-type giant cells, squamous cells, inflammatory cells, calcium deposits, and hemorrhage can be present.^10^^,^^44^ Fine needle aspiration biopsy has previously been described to identify pilomatrixoma; however, this method is not commonly used given the high rates of false positives.^43^^,^^45^^-^47

Complete surgical excision is the most commonly described treatment of pilomatrixoma, regardless of tumor location.^2^^,^^48^^-^50 However, there is no consensus on the optimal margins, excision method, or patient age at the time of excision. Numerous studies have demonstrated a very low recurrence rate with excision alone. Mohs micrographic surgery has also been suggested as a way to achieve tissue-sparing, complete excision with negative margins.^51^ While malignant degeneration and transformation to an aggressive tumor are rare, it is not unheard of, emphasizing the need for complete excision.^52^ More aggressive forms of pilomatrixoma have been described, with such lesions demonstrating local invasiveness, local recurrence, and atypical histologic features, such as high mitotic rate, excessive basaloid proliferation, and discrete nodules distant from the main lesion.^43^^,^^53^^-^55

## CONCLUSIONS

We believe that pilomatrixomas should be included in the differential diagnosis of head and neck masses of infants and children, even in the setting of rapid growth. Treatment is surgical excision with a very low recurrence rate. Preoperative imaging with CT, MRI, or ultrasonography each has some value, but the diagnosis may not be clear until pathologic analysis.
